# Artificial Intelligence-Aided Low Cost and Flexible Graphene Oxide-Based Paper Sensor for Ultraviolet and Sunlight Monitoring

**DOI:** 10.1186/s11671-022-03727-y

**Published:** 2022-09-12

**Authors:** Ahmed Abusultan, Heba Abunahla, Yasmin Halawani, Baker Mohammad, Nahla Alamoodi, Anas Alazzam

**Affiliations:** 1 System on Chip Lab (SoCL), Khalifa University, Abu Dhabi, UAE; 2 Department of Mechanical Engineering, Khalifa University, Abu Dhabi, UAE; 3 Department of Electrical Engineering and Computer Science, Khalifa University, Abu Dhabi, UAE; 4 Research and Innovation Center in Carbon Dioxide and Hydrogen (RICH), Center of Catalysis and Separation, Department of Chemical Engineering, Khalifa University, Abu Dhabi, UAE

**Keywords:** Sunlight, UV, Sensor, Flexible, Reduction, GO, Paper

## Abstract

The adverse effect of ultraviolet (UV) radiation on human beings has sparked intense interest in the development of new sensors to effectively monitor UV and solar exposure. This paper describes a novel low-cost and flexible graphene oxide (GO)-based paper sensor capable of detecting the total amount of UV or sun energy delivered per unit area. GO is incorporated into the structure of standard printing paper, cellulose, via a low-cost fabrication technique. The effect of UV and solar radiation exposure on the GO paper-based sensor is investigated using a simple color change analysis. As a result, users can easily determine the amount of ultraviolet or solar energy received by the sensor using a simple color analysis application. A neural network (ANN) model is also explored to learn the relation between UV color intensity and exposure time, then digitally display the results. The accuracy for the developed ANN reached 96.83%. The disposable, cost-effective, simple, biodegradable, safe, and flexible characteristics of the paper-based UV sensor make it an attractive candidate for a variety of sensing applications. This work provides new vision toward developing highly efficient and fully disposable GO-based photosensors.

**Graphical Abstract:**

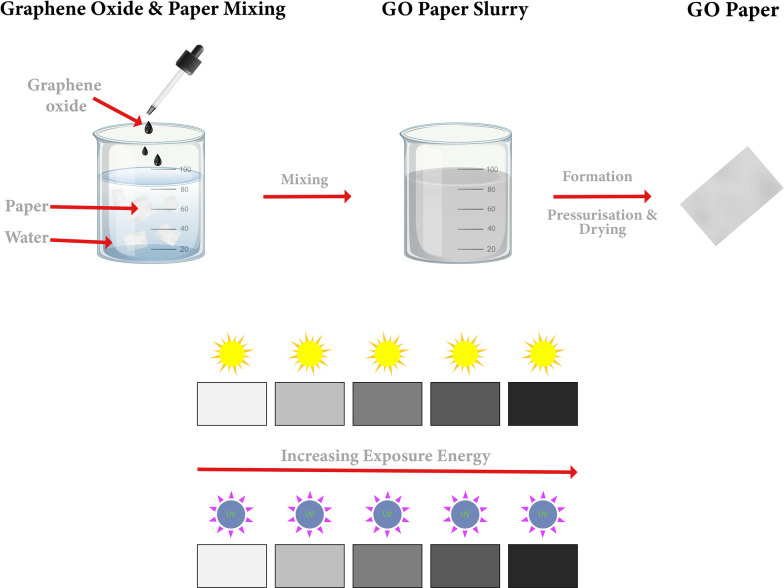

## Introduction

Sunlight, namely UV (wavelengths 290–315 nm), is the principal source of vitamin D for the skin and the body [1]. However, UV overexposure has been linked to the development of various diseases, including skin cancer, benign melanocyte abnormalities (freckles and melanocytic naevi), photoaging (solar elastosis), immune suppression to cataract, photokeratitis, and corneal damage [2–5]. To determine the appropriate exposure dosage to the human body, the solar UV radiation intensity and time of exposure must be monitored. Continuous monitoring of UV exposure at the individual level is critical in naturalistic settings to ensure that exposure is managed safely and effectively [6]. Solar UV exposure can be quantified directly in radiometric units or weighted to account for biologically effective radiation on humans, given that different UV exposures have varying magnitudes of biological effects [7]. As a result, numerous research activities focused on developing UV sensors have been conducted [8]. Photochromic and photoelectric sensors are the most common UV sensors reported in the literature [9]. Photoelectric sensors are composed of a wide bandgap semiconductor integrated within an electric circuit that absorbs and consequently measures UV radiations with a specific bandwidth [10]. On the other hand, photochromic sensors change color in response to UV emission without requiring any electrical input. The photochromic substance goes through a series of photochemical reactions that change it from one photo state to another, where each state has a different color and absorption spectrum. The simplicity of this type of sensors allows the user to detect and capture the UV illumination by the naked eye. Thus, photochromic UV sensors offer direct color signals upon UV radiation without demanding any complex or expensive analytical instruments [8]. Other uses for UV sensors include monitoring ultraviolet radiation exposure to drugs and chemicals that can withstand a certain amount of ultraviolet radiation without being damaged.

Graphene has recently attracted significant attention due to its versatility and unique physical, chemical, and electrical properties, which make it an excellent material for a wide variety of applications [11–14]. Graphene oxide (GO), which is produced by Brodie, Staudenmaier, and Hummer’s techniques that oxidize graphite in the presence of strong acids and oxidants followed by ultrasonic exfoliation of GO sheets [15–17], provides many advantages over other graphene-like materials [18]. For example, GO is commercially available and reasonably priced in comparison to other graphene-similar materials [19]. Additionally, because GO is water soluble, it can be uniformly deposited on a variety of substrates via conventional fabrication techniques such as spin coating, drop casting, and vacuum filtration [15, 20–22]. Moreover, the presence of oxygen functionalities renders it electrically insulating, and its electronic properties can be altered simply by adjusting the GO’s concentration or Carbon-to-Oxygen (C:O) ratio [11, 13, 22, 23]. The reduction of graphene oxide (GO) to reduced graphene oxide (rGO) is accomplished by reducing the oxygen content in order to restore the graphene’s conjugated structure [24]. Thus, rGO resembles graphene but retains oxygen-containing groups, defects, or both. The color of the GO film is a shade of brown, whereas the rGO film is black [25, 26]. Thus, simply by looking at the film, one can approximate the degree of GO reduction by identifying the two distinct colors.

Depending on the target application, GO can be reduced using a variety of methods based on thermal, chemical, or electrical treatments [11, 22, 23]. The work reported in [27] investigated the reduction of GO using the photoreduction approach. The photoreduction of GO to rGO can be classified as a photochemical, photothermal, or a combination of the two processes. The former is more common, where the photogenerated electron hole pairs are the general mechanism for the removal of oxygen groups. Photothermal reduction is based on thermal heating, in which photon energy is converted to thermal energy, thereby deoxidizing GO [24]. Matsumoto et al. [28] showed a simple technique to reduce GO using UV irradiation at room temperature without any photocatalyst. Following this process, the electrical conductivity of GO was significantly increased. Moreover, Li et al. [29] reported a simple method to produce rGO without chemical reductants or other purification processes by exposing GO to irradiation with a Xenon lamp. Furthermore, Smirnov et al. and Plotnikov et al. [30, 31] showed that GO films could be reduced using only UV light. The study observed an increase in the electrical conductivity of the films following UV exposure. It is shown that the GO photoreduction happens, at least partially, if the photon energy exceeds 3.2 eV (*λ* <  ~ 390 nm) [30]. By adding suitable absorbing compounds to GO, the photoreduction of GO can be sensitized to visible light with a wavelength greater than 390 nm. Kamat et al. [32] used TiO_2_ nanoparticles in GO and methanol suspension as a photocatalyst to conduct the photoreduction process by the UV light of a Xenon arc lamp. The majority of semiconductor-based UV sensors currently available require an additional interface to evaluate photocurrent changes and to display the results, which adds complexity and cost.

**Fig. 1:**
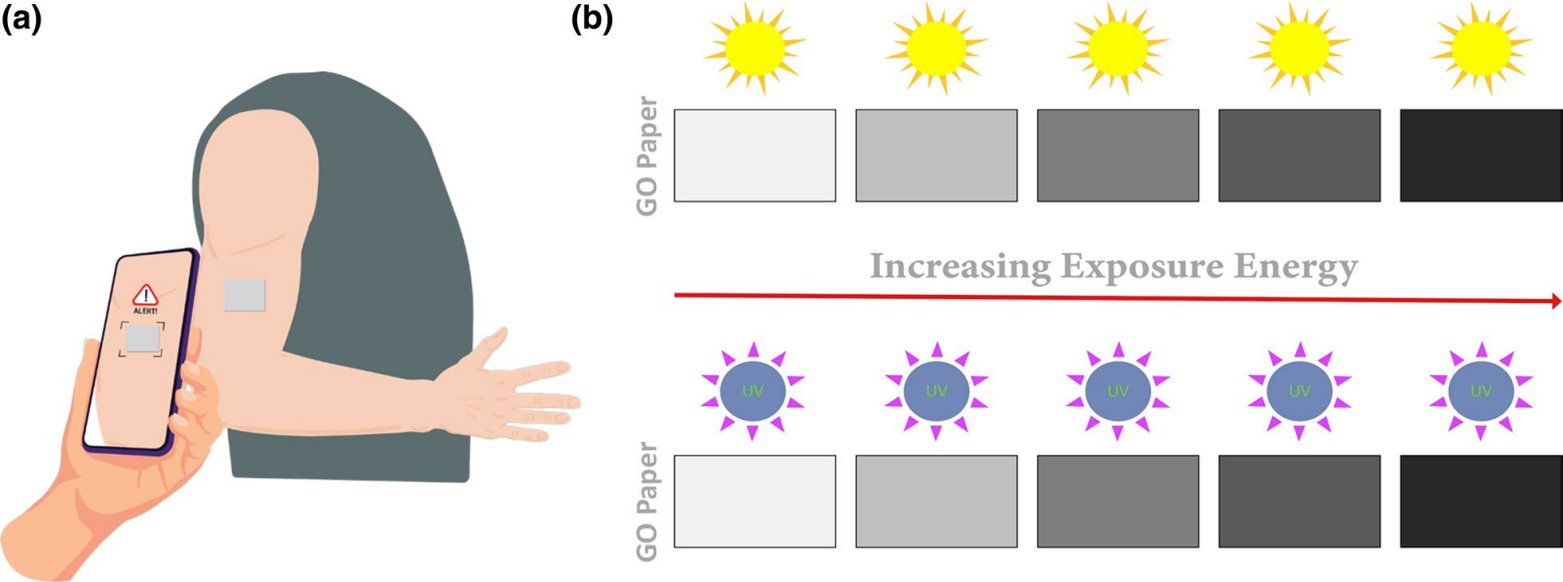
**a** Schematic illustration of the structure and application of the flexible and wearable UV sensor. **b** Schematic diagram for the working principle of the developed sensor. The exposure energy can be monitored or measured through the change in color intensity of the treated samples

The present work describes the development of a novel, disposable, flexible, low-cost, and safe GO-based paper sensor for UV and sunlight monitoring. The developed sensor is entirely composed of paper and is photosensitive due to the incorporation of GO into the structure of the paper. According to the authors’ knowledge, no previous work has been conducted on developing a sensor of this type. The sensor’s proposed method of operation is depicted schematically in Fig. 1a. The device could be worn on the body as a patch, and sunlight or UV exposure can be estimated by observing the color change in the patch after being exposed to sunlight. An artificial intelligence application is developed to estimate sunlight exposure or UV by analyzing color changes and correlating them to exposure energies. GO has "exposure memory" as a result of the reduction process, which means that the changes in its chemical and physical properties are permanent. This memory aids in tracking UV or solar radiation exposure and enables offline measurements at any time after exposure is complete. Higher exposure energies for the GO-paper sensor result in darker color (closer to graphene color), as shown schematically in Fig. 1b. This work provides new insights for the development of efficient fully disposable GO-based photosensors. The rest of the paper is organized as follows. “Materials and methods” section details the novel fabrication of the GO-based paper sensor reported in this work. Also, it discusses the approaches followed in this work to analyze the color intensity of the sensor and to build the ANN model. The “Results and Discussion” section includes four subsections. In “UV Sensing Study” subsection, UV light study is established to investigate UV light effect on the GO paper sensor. This can help in variety of applications that require a certain dose of UV. In “Solar Simulator Study” subsection, solar simulator study is presented to simulate a real case scenario of sun exposure which is not consisting of UV only. This ray generates heat which can also reduce GO. This has a great potential for Sunlight monitoring applications including monitoring working environments for the presence of sunlight to mitigate sun exposure effects and health risks associated. The structural analysis of the novel sensor conducted using field emission scanning electron microscopy (FESEM) is reported in “Structural Characterization” subsection. Then, the results of the built ANN model are discussed in “Artificial Neural Network Classification for Mobile Application” subsection. Finally, concluding points of this work are summarized in “Conclusion” section.

## Materials and methods

### Fabrication Process

**Fig. 2:**
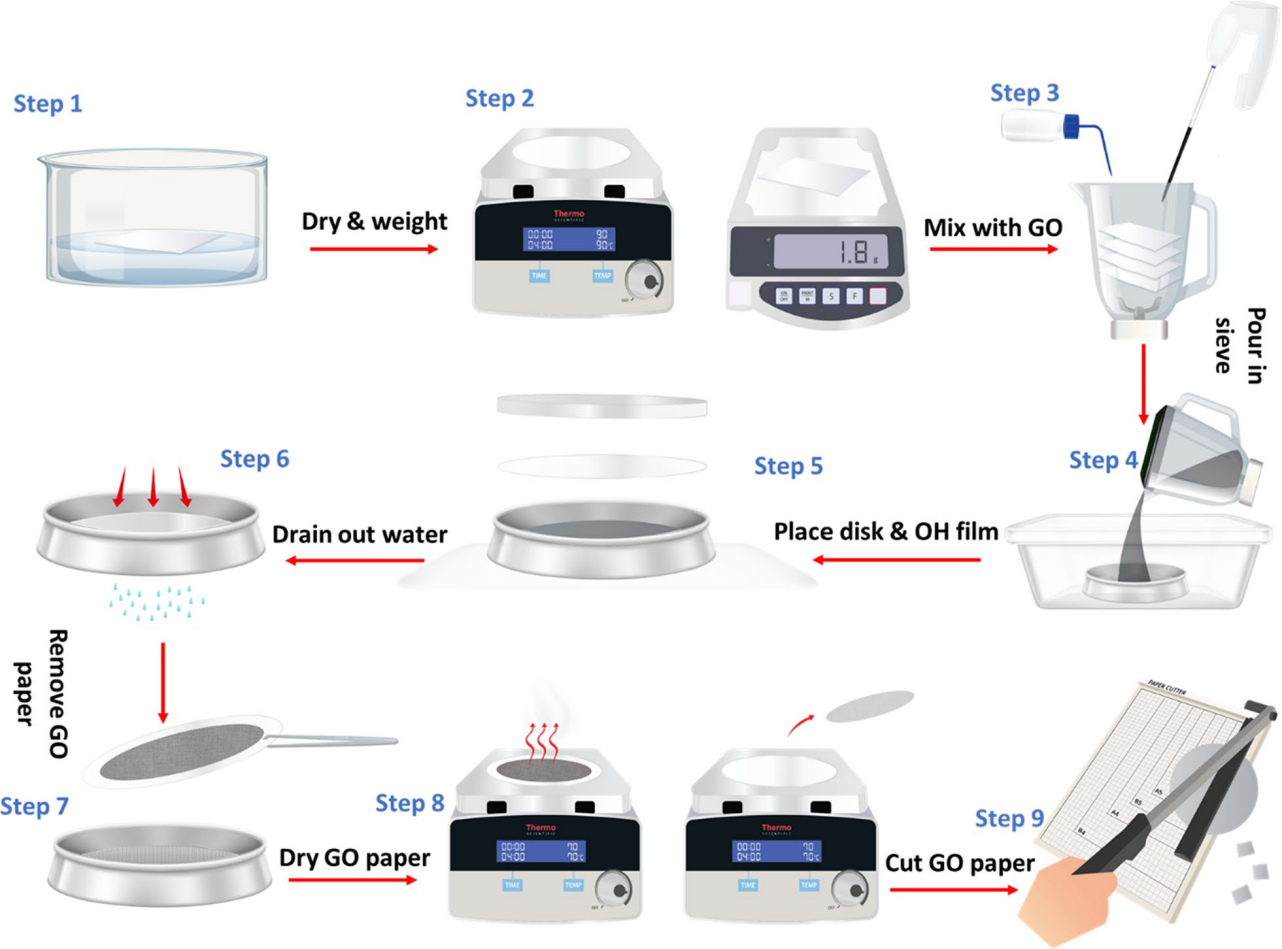
Schematic illustration of the main steps followed to fabricate the GO-based paper sensor reported in this work

The GO-paper sensor is fabricated using a simple and inexpensive fabrication method. Standard copy paper and graphene oxide dispersion in water at a concentration of 4 mg/mL (2.5 wt%, Graphenea, San Sebastian, Spain) are the primary materials used in this fabrication process. A schematic representation for the fabrication process of the GO paper sensor is shown in Fig. 2. In step 1, standard copy paper is washed by soaking it for 24 h in a deionized (DI) water bath to remove any additives and to release the paper fibers. The DI water is changed throughout the process to ensure full removal of the added chemicals. The washed paper is then dried on a hot plate at 90 °C, and the weight of the baked paper is recorded with a weight balance (step 2). The following step involves shredding the papers and adding GO (step 3). The process is accomplished by placing the washed paper in a commercial blender, gradually adding DI water, adding the calculated volume of GO dispersion based on the required GO concentration in the paper (weight of GO to weight of paper ratio), and blending until a smooth slurry is achieved. The mixture is then poured into a 90 μm laboratory sieve inside a container of DI water to create a thin layer of pulp mixture (step 4). After evenly distributing the GO-paper mixture on the sieve's surface, the sieve is slowly lifted from the container. Following that the paper is transferred from the sieve using an overhead projector film, as schematically illustrated (step 5). The transfer step is accomplished by cutting the overhead projector film to the exact dimensions of the sieve and slowly placing it over the GO-paper mixture to avoid air bubbles forming between the film and the paper. A thick plastic disk is then used to apply pressure to the plastic film, allowing the water in the mixture to drain out while it is still in the sieve (step 6). The following step compresses the GO-paper slurry into a sheet, which is then transferred to the plastic film (step 7). The overhead film containing the GO-paper is then placed on a hot plate set to 70 °C until the paper is completely dry and easily detached from the film (step 8). Finally, rectangular pieces (15 × 30 mm) of the fabricated GO-paper are cut to form the sensing devices (step 9).

### Color Intensity Analysis

ImageJ is a public domain Java image processing program inspired by National Institutes of Health Images [33]. The program is used to analyze the change in color intensity of the GO-paper photochromic sensors following exposure to sunlight/UV. In the UV energy study, The GO paper weight–to-weight (w/w %) ratio used is 1%. The UV lamp’s intensity is determined to be (8.7 mW/cm^2^) using an intensity meter (G&R labs, model 202). The studied exposure periods range from 5 min to 60 min with an increment of 5 min. Therefore, the UV energies are calculated based on the intensity and exposure time. In the solar simulator study, the GO concentration is maintained at 1%. In addition, an artificial sunlight simulator (Oriel Sol3A) is used; it features a single lamp design with a single sun output power (100 mW/cm^2^). The exposed samples are analyzed to determine the difference in color intensity between the pre- and post-exposure periods using grayscale function. The color intensity ratio represents the ratio of the grayscale value of the exposed part of the paper sensor to the grayscale value of the unexposed part of the same paper sensor. Gray scale is a set of grayscale values ranging from 0 to 255 for each pixel in a grayscale image. The gray level of each pixel in a color image is calculated using the following formula [34]:

1 $${\text{Grayscale}} = 0.299{\text{R}} + 0.587{\text{G}} + 0.114{\text{B}}$$

where R, G, and B are the red, green, and blue components of the color image. The doses (energies) of both the UV and solar radiations are measured and reported.

### Artificial Neural Network Analysis

Artificial neural networks (ANNs) are widely used models to recognize patterns for classification tasks [35]. ANNs are inspired by the signal processing happening in our neurons which starts by receiving input at the dendrites and summing their weighted contributions at the soma. Then, the information travels the axon until it reaches the synapses. If the value is larger than a certain threshold, then the information will pass to the next neuron. Mapping this phenomenon to mathematical equation

2 $$y = f\left( {\mathop \sum \limits_{i = 1}^{n} w_{i} x_{i} + b_{i} } \right)$$

where $y$ is the output,$f()$ is the activation function for the neuron, $w_{i}$ is the weight of the neuron that contributes to the output, $x_{i}$ is the applied input, and $b_{i}$ is the bias that better fits the input–output relationship. Therefore, the ANN model consists of input, hidden and output layers composed of neurons that mimics the biological counterpart as demonstrated by (2). The size and structure of the network, such as the number of neurons in each layer, and the number of layers, depends on the application and required performance.

Neural network models have training (learning) and inference (testing) phases. During training, and if supervised algorithm is assumed, the model goes through hundreds or thousands of iterations to reduce the error between the applied input and its associated class (label). This happens through backpropagation that adjusts the neuron weight contribution to the output. After a desired accuracy is reached, training stops and the learned weights are now fixed. Through inference phase, the model is now ready to classify unseen input by a single forward pass. The collected GO samples, both exposed and unexposed, are applied as inputs to a simple ANN model for exposure dose classification. The model has been trained on a specific set of data, and then tested on new unseen ones to check for classification accuracy. Detailed discussion on the utilized ANN model, simulation environment, and results are presented in “Artificial Neural Network Classification for Mobile Application” subsection.

## Results and Discussion

### UV Sensing Study

#### Effect of GO Concentration

**Fig. 3:**
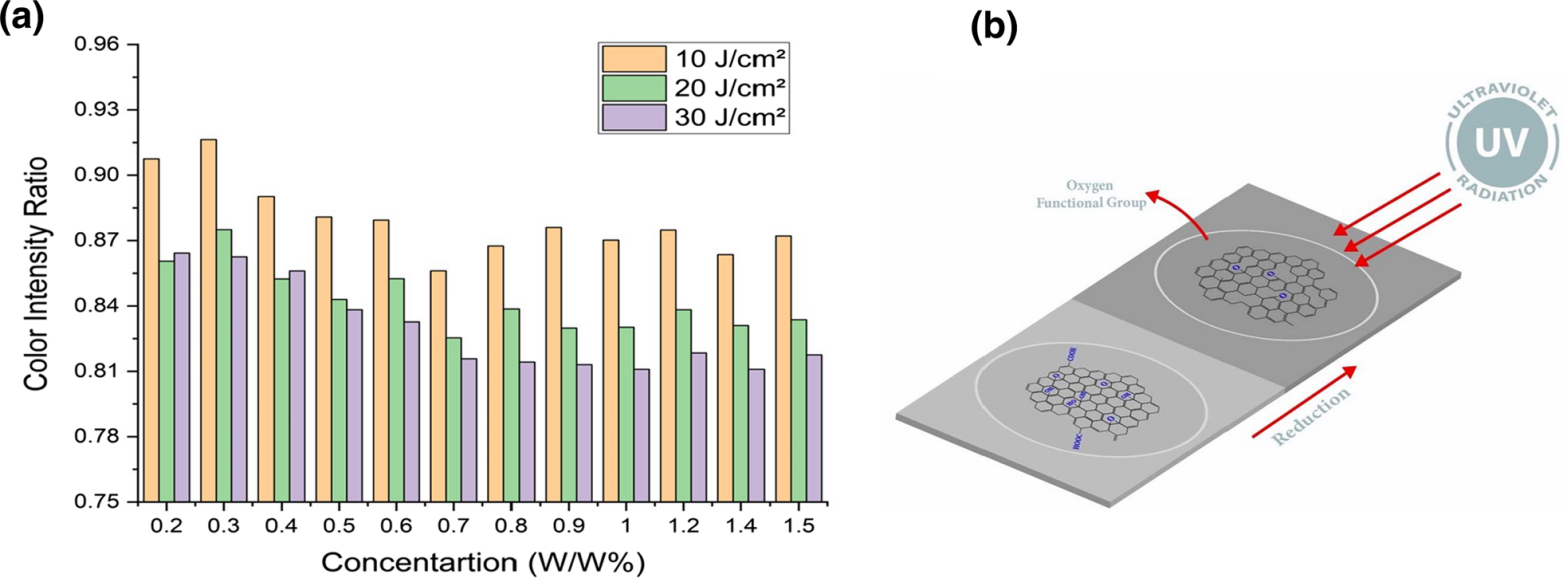
**a** GO concentration-dependent variations in color intensity ratio for samples when exposed to UV doses of 10, 20, and 30 J/cm^2^. **b** Schematic illustrates the reduction mechanism taking place in the fabricated sensor

To investigate the effect of the GO content on the sensor's performance, GO-paper sensors with varying GO concentrations are fabricated. The color intensity ratios of several GO-papers exposed to various doses of energy per unit area are shown in Fig. 3a. The concentration that results in the greatest change in color intensity with respect to the applied energy is preferred for the developed sensor. As shown in the graph, for low GO concentrations, where the amount of GO on the surface is small, there is a large difference in the intensity ratio for the 10 J/cm^2^ compared to 20 J/cm^2^ and 30 J/cm^2^. In addition, lower concentrations reach saturation in terms of color change in shorter periods of time. GO paper samples with 0.2% contain small/insufficient amount of GO. Thus, some characteristics of GO will not be clearly noted [36]. Hence, a dose of 20 J/cm^2^ of UV is sufficient to induce saturating reduction in the sample due to the small amount of GO incorporated in the low concentration samples. As a result, the grayscale values of the 0.2% samples exposed to 20 J/cm^2^ and 30 J/cm^2^ of UV light are similar regardless of the increase of the exposure dosage as shown in Fig. 3a. GO paper (w/w %) less than 1% exhibit a limited range of sensitivity and do not exhibit sufficient changes in color intensity. However, higher (w/w %) ratios have a wider sensitivity range for a wider range of UV exposure. The greater amount of GO gives the ability to achieve the maximum difference in color intensity ratios after UV exposure. However, higher GO concentration can limit the sensor's performance, since GO paper turns dark which contradicts the concept of photochromic sensors. The results for GO concentrations of: 0.8%, 0.9%, 1% and 1.2% are very close to each other and achieve the requirements of the sensor. The GO concentration is maintained at 1% in this work, to increase the sensor's sensitivity and ensure a sufficient change in color. The schematic provided in Fig. 3b shows the working mechanism of the proposed sensor. UV radiation is the main reason of the color change due to the photoreduction process of the GO paper. Therefore, the removal of the oxygen functional groups creates rGO which is darker in terms of color.

#### Effect of UV Energy

**Fig. 4:**
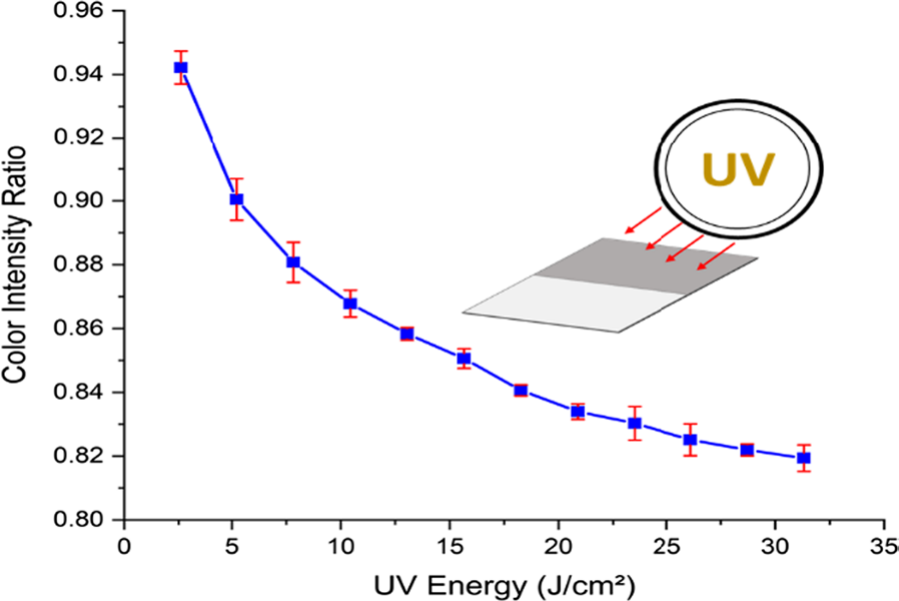
Results of color intensity characterization of the sensors with GO concentration of 1%. The data presented show the UV energy effect on the GO paper sensors

Fresh GO-papers with GO concentration of 1% are exposed to UV for different time durations to achieve different UV energies (doses). The experiment makes use of a UV flood exposure system. As illustrated in Fig. 4, the tested samples are analyzed to determine the difference in color intensity after exposure. The *x*-axis represents the energy of the UV dose in (J/cm^2^), and the *y*-axis represents the color intensity ratio, with 1 representing white and 0 representing black. As can be seen, as the UV energy increases, the color intensity ratios decrease. In other words, the device gradually increases its darkness intensity until it approaches saturation at about 30 J/cm^2^. The color intensity ratios are used to estimate the UV dose using the previously mentioned characterization techniques. This UV photo response indicates that the primary cause of the color change is photoreduction of the GO paper sensors [37].

#### Effect of Heat on Color Intensity

**Fig. 5:**
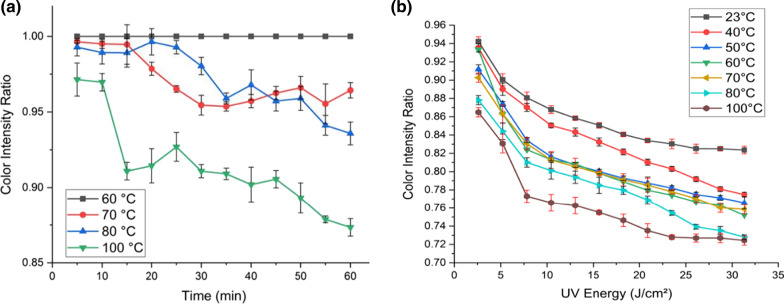
Results of heat effect with GO concentration of 1% at different temperatures on: **a** the color intensity ratio of the sensor under dark conditions. **b** The color intensity ratio of samples at varying temperatures and UV doses. The data presented show the heat effect on the GO paper sensors

The performance of the proposed sensor may be compromised by the presence of heat generated by radiations, as GO can be reduced using thermal methods [38]. As a result, fresh sensors are subjected to heat treatment in order to further investigate the behavior of the fabricated sensors in real-world scenarios. The effect of heat alone (without UV exposure) is investigated by monitoring the change in color intensity of fresh samples over time at various heating temperatures (60–100 °C). As illustrated in Fig. 5a, the color intensity ratio of samples heated to less than 70 °C remains constant at value of 1, indicating that the color of the exposed areas did not change. Specifically, the heating temperature is insufficient to initiate the formation of rGO. However, heating the devices above 70° altered the color intensity ratio, but with no discernible trend. The sensor is intended for use in applications where the temperature does not exceed 50 °C. This temperature range is insufficient for the thermal reduction of GO [23, 39, 40], as the carboxyl groups are expected to be reduced slowly at 100–150 °C [41, 42].

To study the coupled effect of UV exposure and heat, the paper sensors are exposed to UV and heated simultaneously at different temperatures and energy doses. The change in color intensity after UV exposure at various temperatures is depicted in Fig. 5b. As the temperature increases, the intensity ratio gradually decreases. Thus, the addition of heat during UV exposure catalyzes the formation of rGO but UV is the dominant reason of the color intensity variation. The results show that heat accelerates the photoreduction reaction of the GO paper sensors. In the event that the sensor is used in an uncontrolled environment with varying temperatures, the temperature should be incorporated into the AI analyses. On the basis of the obtained color intensity value, the corresponding calibration curve can be used to calculate the UV or sunlight exposure dosage. The proposed sensor has the potential to operate in a variety of UV and sunlight monitoring applications and environments.

#### XRD and Raman Analysis

**Fig. 6:**
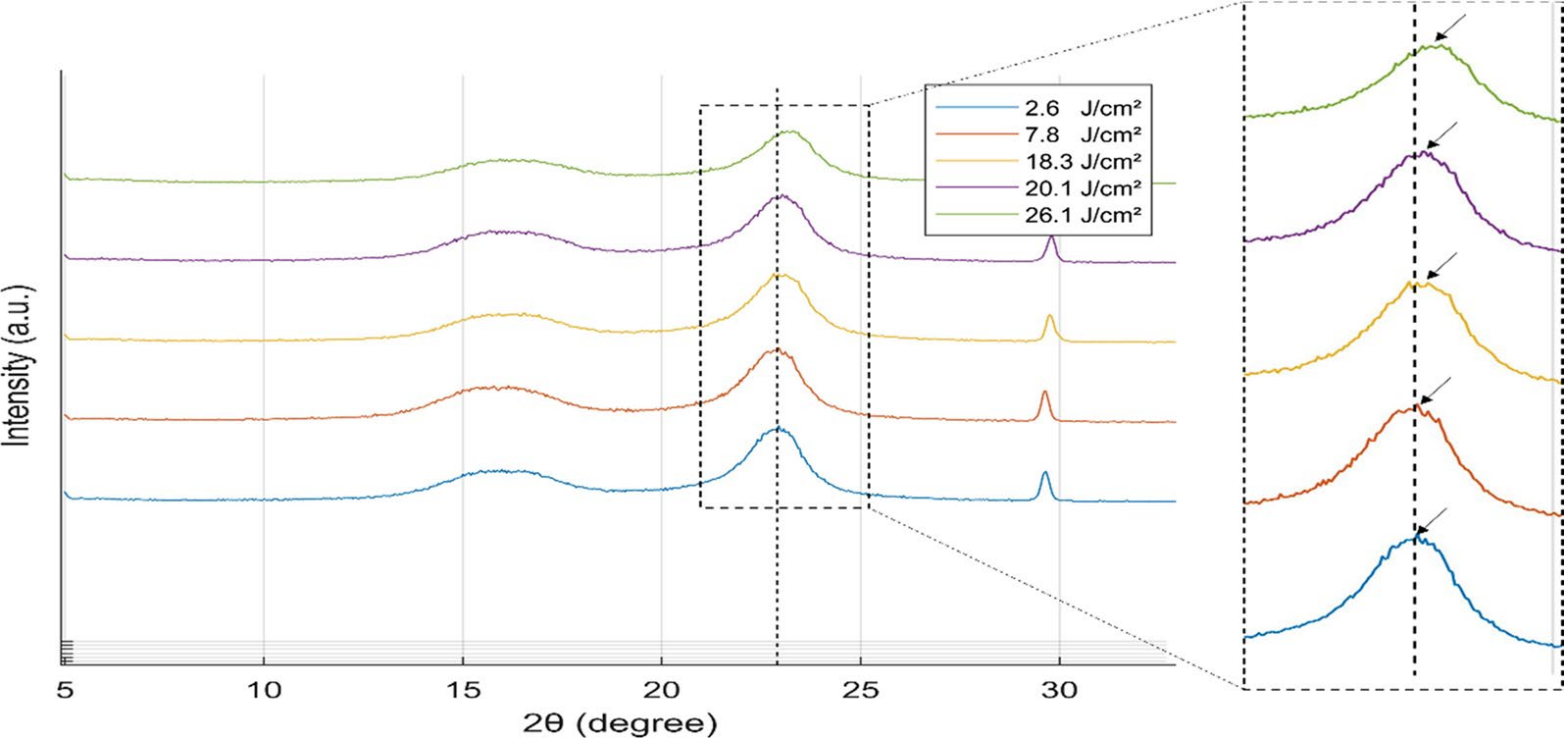
X-ray diffraction (XRD) patterns of fabricated sensors for different UV doses. X-ray diffractometer is operated at 30 kV and 10 mA (*λ* = 1.541 Å) and the scan range of the diffraction angle was set to a range of 5° to 35° with a step size of 0.05°and 0.25 s per step

X-ray diffraction (XRD) analysis is performed on fabricated UV sensors for different UV doses using X-ray diffractometer (Bruker, D2 Phaser). The tests are executed to characterize and calculate the dissimilarity in interlayer spacing (*d*-spacing) in GO papers which gives better comprehension of the change in color intensity. Because of the removal of the oxygen containing functional groups during the reduction process of GO, the interlayer spacing decreases in the graphite layer and results in a graphene-like structure [43]. This causes a change in the chemical and physical properties altering the color intensity of the sensor outer surface. Interlayer spacing is calculated using Bragg’s law: $\lambda = 2d\sin \left( \theta \right)$, where *d* is the interlayer spacing, *θ* is the diffraction angle and *λ* is the X-ray wavelength. The rGO diffraction peak (002) occurs at approximately 2*θ* = 24.5° corresponding to *d*-spacing of 0.36 nm, which shows a slight variance when compared to the standard graphite peak at approximately 2*θ* = 26.4° corresponding to *d*-spacing of around 0.34 nm [44]. The XRD spectrum of the fabricated sensors without reduction shows three peaks at 2*θ* = 16.25°, 22.90° and 29.80° as shown in Fig. 6.

The three peaks correspond to the combination of the peaks of both GO and cellulose, which is the main component found in standard copy paper, and equivalent to the data reported in the literature [45, 46]. However, after the reduction using UV exposure, the peak corresponding to rGO starts to shift to greater angles with the increase of UV dose. This is attributed to the photoreduction of GO and confirming the removal of oxygen-containing functionalities with a decrease in the interplanar distance. In addition, the interlayer spacing is still greater than the interlayer spacing of graphene referring to some remaining oxygen-functionalities groups and defects.

**Fig. 7:**
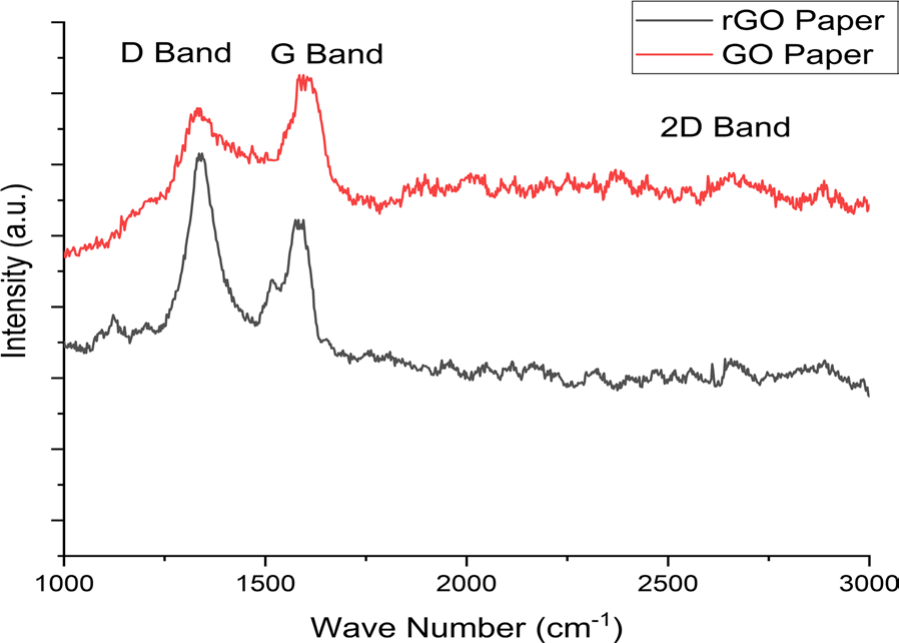
Raman spectra of fabricated devices before (black curve) and after (red curve) reduction from 1000 to 3000 cm.^−1^

Figure 7 shows the Raman spectra for a paper sensor before reduction (GO) and after reduction (rGO). Two dominant vibrations are present in the range of 1000 and 3000 cm^−1^ for both devices. The D vibration band is associated with the breathing mode of j-photons of A_1g_ symmetry [47]. The D band appears at 1330.6 cm^−1^ and 1335.6 cm^−1^ for GO and rGO paper, respectively. The intensity of the D band increases with the number of defects in the graphene plane. Therefore, upon reduction, the D band increases as the removal of the oxygen functionalities causes the formation of the defects in the graphene plane. The G vibration band appeared at 1584.8.5 cm^−1^ for GO paper and 1575.1 cm^−1^ for rGO paper, which describes the first order E_2g_ optical mode of the C–C double bond of the graphite plate [47]. The position of the G band shifts to a higher wave number when a higher degree of graphene oxidation is presented as GO, and the reduction process causes the G band to shift to a lower frequency, as shown in Fig. 7 for the rGO paper [47–49]. The D and G bands represent the disorder and tangential bands, respectively. Moreover, the 2D band is observed at 2652 cm^−1^ for GO paper and 2641.5 cm^−1^ for rGO paper. This band is sensitive to the stacking of graphene layers, and therefore it can be used to determine the layers of graphene. The shifting of the 2D band after reduction is due to the lower number of oxygen functional groups, which leads to rGO stacking. In addition, the *I*_D_/*I*_G_ ratio can be used to symbolize the defects in the GO structure. The *I*_D_/*I*_G_ ratio increases from 0.99 for GO to 1.02 for rGO. This is mainly because of the decreased size of *sp*^2^ domains and the restoration of *sp*^2^ carbon after reduction.

### Solar Simulator Study

#### Effect of GO Concentration

**Fig. 8:**
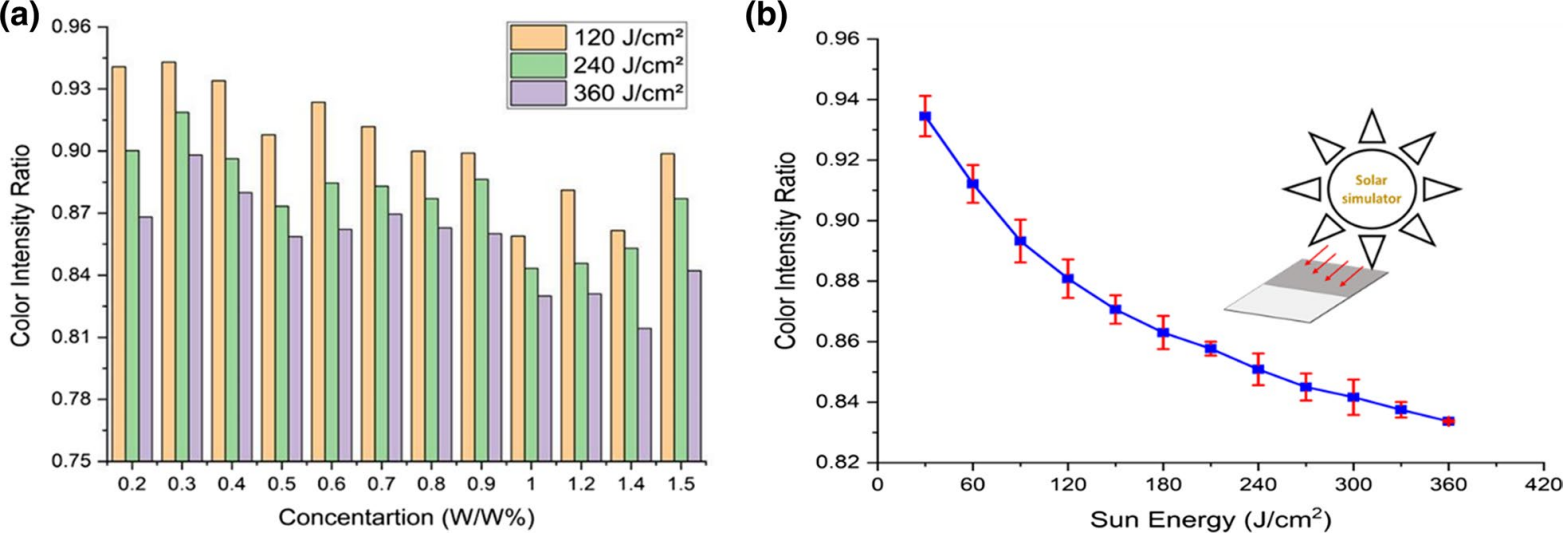
**a** Difference in color intensity for different GO content solar radiation exposed samples. **b** Results of color intensity characterization of the sensors with GO concentration of 1%. The data presented show the exposure energy effect on the GO paper sensors

The process used to evaluate the influence of UV light on the sensor is repeated to determine the effect of the GO content in the GO-paper on the sensor's performance under solar radiation. GO-paper sensors with varying GO concentrations are fabricated. In this investigation, the color intensity ratios of the samples prior to and following exposure are depicted in Fig. 8a. As illustrated in the graph, the GO paper (GO w/w % concentration) ratio at 1% exhibits a useful range of sensitivity for a wide range of sun exposure. However, concentrations of GO in the vicinity of this ratio had no noticeable effect on the sensor's sensitivity.

#### Effect of Solar Energy

To investigate the sensor performance, the GO-paper sensors are exposed to sunlight for varying durations. Similarly, to the UV energy study, the exposed samples are evaluated to determine the difference in color intensity between the pre- and post-exposure states. Figure 8b illustrates the variation in color intensity between the pre- and post-exposure states for different samples. The *x*-axis represents the sun energy in J/cm^2^, while the *y*-axis represents the difference in color intensity for the samples after exposure when compared to the same before exposure. As illustrated in the graph, as the exposure energy increases, the color intensity ratios drop due to the reduction of GO to rGO. These results highlight the remarkable functionality and sensitivity of photo sensing applications utilizing flexible and disposable sensors.

#### XRD Analysis

**Fig. 9:**
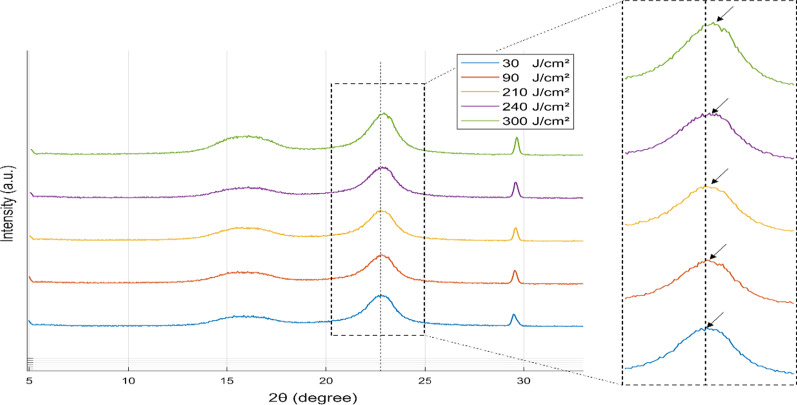
X-ray diffraction (XRD) patterns of fabricated sensors for different solar doses. X-ray diffractometer is operated at 30 kV and 10 mA (*λ* = 1.541 Å) and the scan range of the diffraction angle was set to a range of 5° to 35° with a step size of 0.05°and 0.25 s per step

The peak corresponding to rGO, in the XRD spectrum of the fabricated sensors, exhibits shifting to greater angles with the increase of exposure duration of solar simulator. After one hour of solar radiation exposure, the rGO peak shifted from 2*θ* = 22.85° to 2*θ* = 23.15°, as illustrated in Fig. 9. This shifting is attributed to the redaction process and the removal of the oxygen groups which decreases the interlayer spacing as explained previously. However, the interlayer spacing is also still greater than the interlayer spacing of graphene after the solar radiation exposure, because of the remaining oxygen-functionalities groups and defects.

Comparing the results presented in Figs. 6 and 9, under UV exposure, the rGO diffraction peak (002) occurs at approximately 2*θ* = 22.90°, corresponding to a *d*-spacing of 0.396 nm. The peak shifted after reduction to 2*θ* = 23.53°, corresponding to a *d*-spacing of 0.379 nm. Under solar radiation, the rGO diffraction peak (002) occurs at approximately 2*θ* = 22.85°, corresponding to a *d*-spacing of 0.390 nm. The peak shifted to 2*θ* = 23.15°, which corresponds to a *d*-spacing of 0.385 nm, following reduction by solar radiation. This shift in 2*θ* in both figures is a result of the redaction procedure and the removal of oxygen groups, which decreases the interlayer spacing, as previously described in the manuscript. Compared to exposure to a solar simulator, UV exposure has a greater impact on the interlayer spacing of samples.

### Structural Characterization

**Fig. 10:**
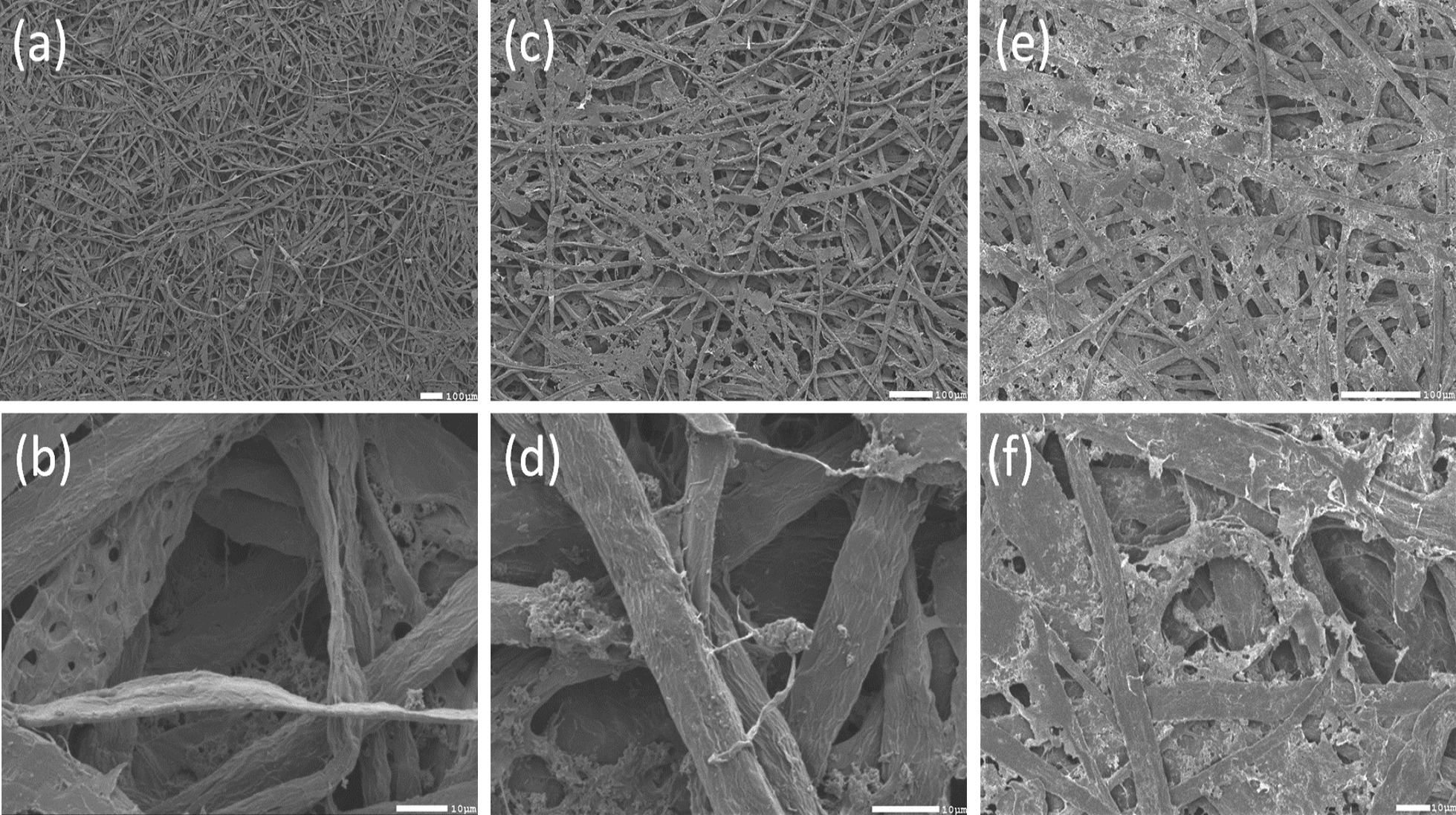
FESEM for: **a** fabricated plain paper without GO (magnification: 55 ×, accelerating voltage: 5 kV, working distance: 8 mm). **b** Fabricated plain paper without GO (magnification: 1300 ×, accelerating voltage: 5 kV, working distance: 8 mm). **c** Top surface of the GO paper sensor before photoreduction (magnification: 110 ×, accelerating voltage: 5 kV, working distance: 8 mm). **d** Top surface of the GO paper sensor before photoreduction (magnification: 1700 ×, accelerating voltage: 5 kV, working distance: 8 mm). **e** Top surface of the GO paper sensor after photoreduction (magnification: 200 ×, accelerating voltage: 5 kV, working distance: 8 mm). **f** Top surface of the GO paper sensor after photoreduction (magnification: 850 ×, accelerating voltage: 5 kV, working distance: 8 mm)

The morphologies of GO-paper sensor and conventional printing paper (without GO) fabricated using the same process are compared. Both samples are analyzed using field emission scanning electron microscopy (FESEM). The fibers of the fabricated plain paper are depicted in Fig. 10a, b. The paper's structure appears to be composed of interwoven cellulose microfibers that are forcefully squeezed together, resulting in non-uniform surfaces that dictate the paper's surface roughness. The GO flakes before photoreduction are embedded in the fibers of the paper as shown in Fig. 10c, d. After UV treatment, roughness and morphology change due to the formation of rGO from GO which compacts the material by the removal of the Oxygen groups [50]. All flakes seem smooth and form aggregates as shown in Fig. 10e to f.

### Artificial Neural Network Classification for Mobile Application

**Fig. 11:**
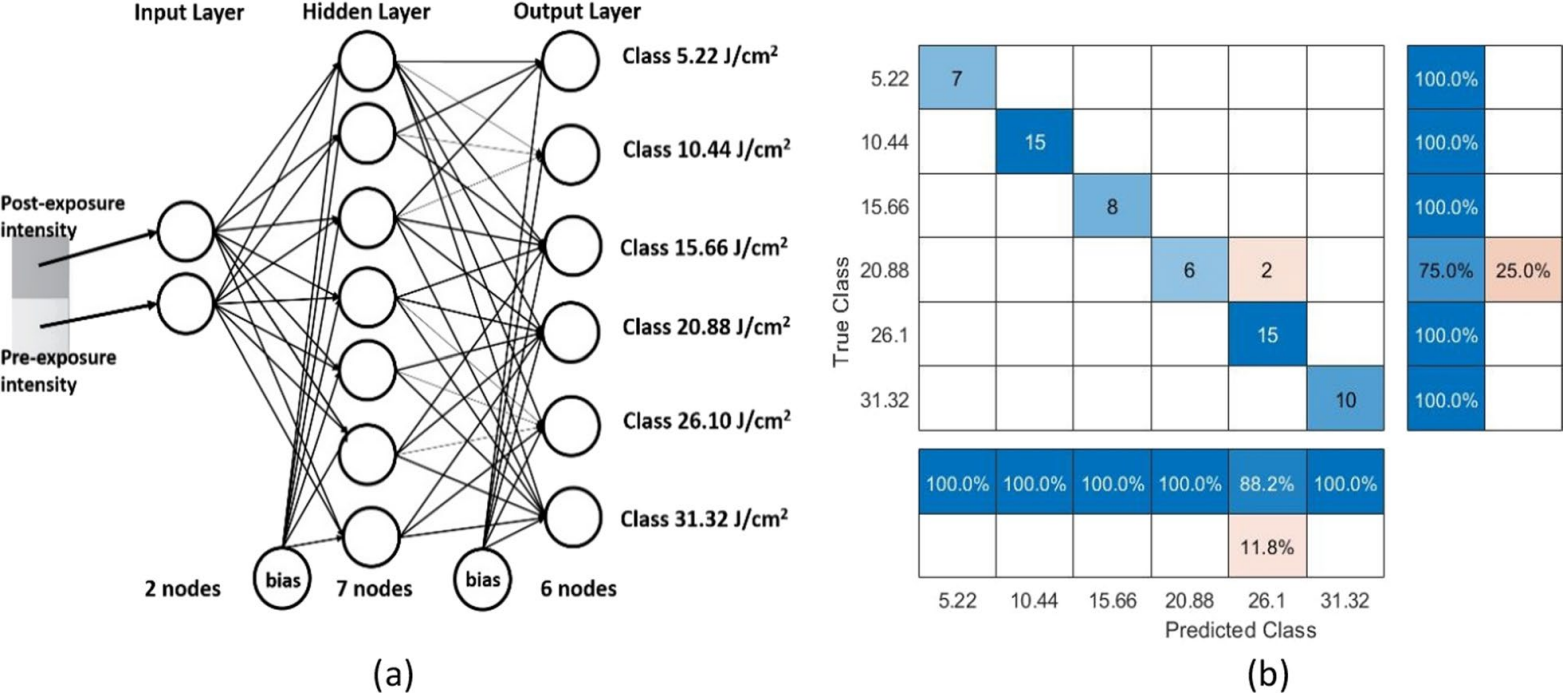
**a** Graph representation of the perceptron with two input nodes, 7 hidden neurons, and 6 output classes. **b** Confusion Matrix chart that presents the correctly classified exposure intensities on the diagonal. It also demonstrates the recall and precision bars of how well the ANN model performs

A mobile application that utilizes artificial neural network (ANN) model can be used to directly predict the exposure dose of the flexible and wearable UV sensor. In this work, a simple ANN is built to learn the relation between the unexposed GO paper sample and the sun-exposed ones with respect to the exposure dose. As shown in Fig. 11a, the inputs to the ANN are the unexposed and exposed pixel intensity values. The output represents the exposure dose which is one of six classes ranging from 5.22 to 31.32 J/cm^2^. The collected input samples from 313 GO paper photochromic sensors were divided into 80% training and 20% testing. ANN training and testing are performed using MATLAB R2019a. The classification accuracy reaches 96.83%. Figure 11b presents the confusion matrix chart of the predicted versus actual classes. The blue diagonal values resemble the classes that have been correctly classified. Recall and precision are ANN model performance metrics presented by the lower and right-sided bars, respectively.

For the neural network (NN) application proposed in this work, it is assumed that an image is captured via any mobile application. The captured image is then pre-processed and divided into two regions. The grayscale intensity of each region is then extracted. The intensity information is obtained by calculating the mode (the value that appears most frequently in a set of data values) of the segmented regions to ensure that outliers have a minimal effect on the intensity value. Convolutional neural networks (CNNs), as opposed to ANNs, are more computationally demanding and have a larger number of adjustable parameters when classifying images. In order for ANN to classify images, the 2D input image must first be converted into a 1D vector, which significantly increases the number of input nodes (parameters) for ANN. In addition, CNNs would need more training data to successfully classify images. We desired a straightforward, lightweight classification method, so it was based on the intensity values of the segmented regions and ANN.

It is worth mentioning that there are several machine learning classification techniques such as NN, support vector machine (SVM) and k-nearest neighbor (KNN). Each technique comes with its pros, cons, and own challenges [51–56]. NN are widely used classifiers and have provided an acceptable accuracy result for the application proposed in this work.

Table 1 summarizes the important features of the available flexible UV/sunlight photodetectors. As shown, the sensor presented in this work has the simplest structure as the GO is incorporated into the paper and no need for substrate material to be used in the device structure. Also, it is the first and the only photosensor that utilizes the usage of AI Aided application. Thus, this work demonstrates novel approach toward developing smart disposable, low-cost, simple, biodegradable, and flexible GO-based paper UV/sunlight sensor.

**Table 1:** Comparison of important features of available flexible UV/Sunlight sensors

References	Material/hybrid	Substrate	Fabrication technique	Biodegradability	Cumulative dose	Type of detection	AI-aided
[57]	Graphene/ZnO	Paper	Direct writing	Yes	No	Photoelectric	No
[58]	ZnO/carbon	Paper	Screen printing and Miura origami folding method	Yes	No	Photoelectric	No
[59]	rGO/ZnO	Mica	Femtosecond laser direct writing (FsLDW)	Yes	No	Photoelectric	No
[60]	TiO_2_/Graphene	Parylene-C/Silicon	Photolithography and Spraying	No	No	Photoelectric	No
[61]	Prussian Blue/TiO_2_	Polyethylene terephthalate (PET)/ indium-tin oxide (ITO)	Cyclic voltammetry Scan, anodization and annealing	–	No	Photochromic	No
[62]	C1H/MG, DPIC/TB^a^	Polyvinyl Butyral (PVB)	Spin coating	No	Yes	Photochromic	No
[63]	PMA/LA^b^	Filter paper	Pen writing	–	Yes	Photochromic	No
Current work	GO/Paper	NA	Blending GO and paper through novel fabrication process	Yes	Yes	Photochromic	Yes

^a^Chloral hydrate/malachite green/diphenyliodomium chloride/ thymol blue

^b^Phosphomolybdic acid/lactic acid

## Conclusion

A novel flexible and disposable GO paper-based UV and solar sensor has been developed. A low-cost and simple fabrication method was used to develop the sensor. An AI application was developed to characterize the sensor, which measures the exact change in color caused by UV and solar exposure. The results verified that the sensor could detect a long range of UV and sunlight exposures. The UV/solar sensor was used for offline measurement of exposure to solar radiation and/or UV radiation by the irreversible reduction of graphene oxide upon exposure. The advantages of being flexible and made of low-cost fabricated GO paper make it a great potential candidate for various applications, especially in the pharmaceutical and drug industry or health and safety field. An XRD analysis was conducted on the fabricated sensors during different UV doses and exposure durations. The results showed reduced interlayer spacing of the graphite layers, which confirmed the reduction process and provided a better understanding of the change in chemical and physical properties. Furthermore, the morphology of the device was studied using SEM to obtain the device microphotographs. An ANN model was also used to learn the relationship between the sensor’s color intensity ratio and UV or solar exposure time, with the results being digitally displayed. The study reported here verified the functionality of the proposed disposable, flexible, low-cost, and safe sensor providing both visual detection and real-time monitoring, and it can be optimized to be compatible with the desired application.

## Data Availability

All data generated or analyzed during this study are included in this published article.
